# Incidence of falls and fall-related injuries and their predictive factors in frail older persons with cancer: a multicenter study

**DOI:** 10.1186/s12877-022-03574-7

**Published:** 2022-11-19

**Authors:** Cindy Kenis, Lore Decoster, Johan Flamaing, Philip R. Debruyne, Inge De Groof, Christian Focan, Frank Cornélis, Vincent Verschaeve, Christian Bachmann, Dominique Bron, Heidi Van den Bulck, Dirk Schrijvers, Christine Langenaeken, Pol Specenier, Guy Jerusalem, Jean-Philippe Praet, Jessie De Cock, Jean-Pierre Lobelle, Hans Wildiers, Koen Milisen

**Affiliations:** 1 Department of General Medical Oncology, University Hospitals Leuven, Leuven, Belgium; 2 Department of Geriatric Medicine, University Hospitals Leuven, Leuven, Belgium; 3 Department of Public Health and Primary Care, Academic Centre for Nursing and Midwifery, KU Leuven - University of Leuven, Leuven, Belgium; 4 Department of Medical Oncology, Oncologisch Centrum, Universitair Ziekenhuis Brussel, Vrije Universiteit Brussel, Brussels, Belgium; 5 Department of Public Health and Primary Care, Gerontology and Geriatrics, KU Leuven – University of Leuven, Leuven, Belgium; 6 Department of Medical Oncology, Kortrijk Cancer Centre, AZ Groeninge, Kortrijk, Belgium & Medical Technology Research Centre (MTRC), School of Life Sciences, Faculty of Science and Engineering, Anglia Ruskin University, Cambridge, UK; 7 Department of Geriatric Medicine, St. Augustinus, Wilrijk, Belgium; 8 Department of Oncology, Clinique CHC Montlégia, Liège, Belgium; 9 Department of Medical Oncology, Cliniques Universitaires Saint-Luc, UCLouvain, Brussels, Belgium; 10 Department of Medical Oncology, GHDC Grand Hôpital de Charleroi, Charleroi, Belgium; 11 Department of Geriatric Medicine, AZ Sint-Lucas, Ghent, Belgium; 12 Department of Hematology, ULB Institut Jules Bordet, Brussels, Belgium; 13 Department of Medical Oncology, Imelda hospital, Bonheiden, Belgium; 14 Department of Medical Oncology, ZNA Middelheim, Antwerp, Belgium; 15 Department Medical Oncology, AZ Klina, Brasschaat, Belgium; 16 Department of Medical Oncology, University Hospital Antwerp, Antwerp, Belgium; 17 Department of Medical Oncology, Centre Hospitalier Universitaire Sart Tilman and Liege University, Liege, Belgium; 18 Department of Geriatric Medicine, CHU St-Pierre, Free Universities Brussels, Brussels, Belgium; 19 Department of Geriatric Medicine, Universitair Ziekenhuis Brussel, Vrije Universiteit Brussel, Brussels, Belgium; 20 Department of Oncology, Laboratory of Experimental Oncology, KU Leuven – University of Leuven, Leuven, Belgium; 21 Department of Oncology, KU Leuven – University of Leuven, Leuven, Belgium

**Keywords:** Older persons, Cancer, Frailty, Falls, Fall-related injuries, Geriatric assessment

## Abstract

**Background:**

Falls and fall-related injuries are a major public health problem. Data on falls in older persons with cancer is limited and robust data on falls within those with a frailty profile are missing. The aim of this study is to investigate the incidence and predictive factors for falls and fall-related injuries in frail older persons with cancer.

**Methods:**

This study is a secondary data analysis from data previously collected in a large prospective multicenter observational cohort study in older persons with cancer in 22 Belgian hospitals (November 2012–February 2015). Patients ≥70 years with a malignant tumor and a frailty profile based on an abnormal G8 score were included upon treatment decision and evaluated with a Geriatric Assessment (GA). At follow-up, data on falls and fall-related injuries were documented.

**Results:**

At baseline 2141 (37.2%) of 5759 included patients reported at least one fall in the past 12 months, 1427 patients (66.7%) sustained an injury. Fall-related data of 3681 patients were available at follow-up and at least one fall was reported by 769 patients (20.9%) at follow-up, of whom 289 (37.6%) fell more than once and a fall-related injury was reported by 484 patients (62.9%). Fear of falling was reported in 47.4% of the patients at baseline and in 55.6% of the patients at follow-up. In multivariable analysis, sex and falls history in the past 12 months were predictive factors for both falls and fall-related injuries at follow-up. Other predictive factors for falls, were risk for depression, cognitive impairment, dependency in activities of daily living, fear of falling, and use of professional home care.

**Conclusion:**

Given the high number of falls and fall-related injuries and high prevalence of fear of falling, multifactorial falls risk assessment and management programs should be integrated in the care of frail older persons with cancer. Further studies with long-term follow-up, subsequent impact on cancer treatment and interventions for fall prevention, and integration of other important topics like medication and circumstances of a fall, are warranted.

**Trial registration:**

B322201215495.

## Introduction

Falls are a major problem among the aging population. A fall is defined by ProFouND (The Prevention of Falls Network for Dissemination) as “an unexpected event that causes the person to fall to the ground, floor or a lower level” [1, 2]. According to the World Health Organization (WHO), 28–35% of people over the age of 65 fall at least once a year. This number rises to 32–42% in people over the age of 70 [3]. Approximately half of these persons fall more than once a year [4].

The etiology of falls is complex because biological, behavioral, environmental, and socioeconomic factors play an important role [3]. Consequences of fall incidents can occur on a physical, psychosocial, and financial level. For example, 5–10% of fall incidents lead to serious injury including fractures, tissue damage, or head trauma [5]. On a psychosocial level, fear of falling, reduced social interaction, and a decrease in the quality of life can occur. Fall and fall-related injuries also have financial consequences. In 2015, the direct medical costs for fatal and non-fatal falls are estimated to be $50 billion in the USA. Almost 99% of these costs are attributable to non-fatal falls [6]. In addition, the economic burden of falls seems to be sex dependent, with older females requiring greater healthcare use than older males after a fall.

Little research on a large scale has been done on fall problems in older persons with cancer, even though cancer is a disease of aging. Due to the aging of the population, a 67% increase in cancer incidence for older patients is expected in the USA [7]. Worldwide, 26.4 million new cancer diagnoses are expected every year [8]. In the literature the incidence of self-reported falls in this population varies from 17.6 to 35.8%, depending on the (sub) population of patients with cancer studied and the period of follow-up [9–11]. Although there is inconsistency whether falls are more common in older adults with cancer than without, two studies showed that older persons with cancer are 16–17% more likely to have a fall incident compared to those without cancer [12, 13].

The etiology of falls in older persons with cancer is similar to that of the general older population. In addition, there are specific disease-related risk factors including fatigue, depression, pain, malnutrition, anemia, metastases, and certain chemotherapeutic agents [14].

Within the population of older persons with cancer, a further distinction can be made between older persons with or without a frailty profile. For the concept of frailty, however, there is no consensus yet on a clear definition. In the literature, frailty is often described as an abnormal physiological condition that makes a person more sensitive to stressors and increases the risk of negative health outcomes [15, 16]. In previous research a cancer history and frailty were independently associated, for the most part resulting from high prevalence of geriatric syndromes like falls [12, 17]. Cancer treatments like surgery, systemic therapy, and radiotherapy are possible stressors that can cause the transition from a robust state to a frail state of older patients [18, 19].

The physical consequences of falls are significantly higher in older persons with cancer than in the general older population. For example, 29 to 74% of fall incidents in older persons with cancer result in serious injuries. According to Mohile et al., this can be explained by increased frailty [17]. A fall in older persons with cancer can cause delays or complications in treatment, and can have an impact on the course of the disease, care planning, and prognosis [20, 21].

Current literature regarding falls in older patients with cancer relies on a patient population that integrates patients with and without a frailty profile or doesn’t report any data related to a frailty profile. Robust data regarding falls in older patients with cancer and a frailty profile are missing. However, knowledge about this problem is important because this frailty, in combination with cancer, entails additional risks such as an increased risk for hospitalization and/or mortality, which should be taken into account in the older population [12].

This study aims to investigate the incidence of falls and fall-related injuries in older persons with cancer and a frailty profile and to investigate the predictive factors of these fall incidents and fall-related injuries.

## Patients and methods

This secondary data analysis, focusing on falls and fall-related injuries at baseline and approximately 3 months follow-up, uses data previously collected in a large prospective multicenter observational cohort study in older persons with cancer [22].

### Study design and population

The population of older persons with cancer (both in- and outpatients) was approached between November 2012 and February 2015, spread over 22 Belgian hospitals (8 academic and 14 non-academic hospitals). The inclusion criteria were 70 years or older and the presence of a malignancy (solid tumor or hematologic malignancy). Patients were included at diagnosis or at disease progression / relapse (when a change in therapeutic strategy was considered). Patients underwent a frailty screening using the G8 screening tool followed by a geriatric assessment (GA) if the G8 score was ≤14 out of 17 indicating the presence of a frailty profile [22, 23]. Follow-up was foreseen at approximately 3 months (further described as ‘at follow-up’) [22]. The study was approved by the ethics committee of all participating centers (B322201215495).

### Patient, socio-demographic and clinical characteristics

The following patient, socio-demographic and clinical characteristics were collected: age, sex, social data (e.g. living situation, professional home care), tumor-specific data (e.g. new diagnosis vs. progression / relapse; solid tumor vs. hematological malignancy), comorbidities using the Charlson Comorbidity index (CCI) (no comorbidities vs. comorbidities score ≥ 1/37) [24], polypharmacy by the number of drugs taken the week before inclusion (number of drugs < 5 vs. ≥5) [25], and the Eastern Cooperative Oncology Group - Performance Status (ECOG-PS) [26].

### Geriatric assessment

The following geriatric domains were assessed within the GA: functional status (FS) by activities of daily living (ADL) (independent score 6 vs. dependent score ≥ 7) [27] and instrumental activities of daily living (iADL) (male: independent score 5 vs. dependent score < 5; female: independent score 8 vs. dependent score < 8 )[28]^,^ the presence of pain and fatigue using a visual analogue score (VAS) (no pain versus presence of pain VAS ≥1/10 and no fatigue vs. presence of fatigue VAS ≥1/10) [29, 30], cognition by mini mental state examination (MMSE) (normal cognition score ≥ 24/30 vs. cognitive impairment score < 24/30) [31], mood status using the geriatric depression scale (GDS-15) (no risk for depression score < 5/15 vs. risk for depression score ≥ 5/15) [32], and nutritional status using the mini nutritional assessment – short form (MNA-SF) (no risk of malnutrition (score ≥ 12) vs. risk of malnutrition (score 8–11) vs. malnourished (score ≤ 7)) [33, 34].

### Falls, fall-related injuries and fear of falling

At baseline all included patients were asked whether they had fallen in the past 12 months and whether they had fall-related injuries (minor / major) [22]. We subsequently divided patients into two groups, namely non-fallers (no falls) and fallers (presence of ≥1 fall). We further divided the fallers into single fallers (=1 fall) or recurrent fallers (≥2 falls) [35]. In addition, fall-related injuries were documented and categorized in minor and major injuries. Minor injuries were scrapes and scratches, bruises, and superficial wounds that required no or minimal medical assistance. Major injuries were sprains, severe soft-tissue bruises, severe head injuries, distortion or dislocation of the joints, contusions, lacerations, loss of consciousness, and fractures [36, 37].

At approximately 3 months follow-up, falls and fall-related injuries were recorded again by asking the patients whether they had fallen and had a fall-related injury as a consequence during the follow-up period.

Finally, fear of falling (i.e. never, sometimes, often, always) was assessed at both time points [38, 39].

### Statistical analysis

We used descriptive statistics (mean, median, standard deviation, range for continues data, and frequency for categorical data) to describe patient characteristics and calculated the 95% confidence intervals. Proportions were compared using a Chi square test. Statistical techniques for handling missing data were not used. Statistical significance was considered at *p* ≤ 0.05.

Univariable logistic regressions were conducted with non-fallers versus fallers (≥1 fall), fallers without injuries versus fallers with injuries (minor and major combined) as the dependent variables. The independent baseline variables were: age, sex, characteristics of the tumor (solid vs. hematologic malignancy; new diagnosis vs. relapse/progression), CCI, polypharmacy, ECOG-PS, living situation, professional home care, ADL, IADL, falls history in the past 12 months, fear of falling, VAS for fatigue, VAS for pain, MMSE, GDS, and MNA-SF. These variables were dichotomized, except for the variable ‘age’, which was divided into four categories (i.e. 70–74, 75–79, 80–84, ≥85).

After univariable analyses, we conducted multivariable logistic regressions in order to explore the relationship between the dependent variables (non-fallers versus fallers, and fallers without injuries versus fallers with injuries), and patients’ baseline significant (*p* ≤ 0.05) characteristics identified in the univariable analyses.

Multivariable logistic regressions were conducted both without selection and with stepwise selection. Data of the regressions with selection are shown. The *p*-values to enter and stay in the model were 0.05. In case of only two significant variables, the multivariable logistic regression was done without selection.

Multicollinearity of the independent variables was investigated with the variance inflation factors (VIF). If the VIF was < 3, absence of multicollinearity was concluded. All statistical analyses were performed with SPSS 25.0 or with SAS v.9.4 software.

## Results

### Patient, socio-demographic and clinical characteristics

**Fig. 1:**
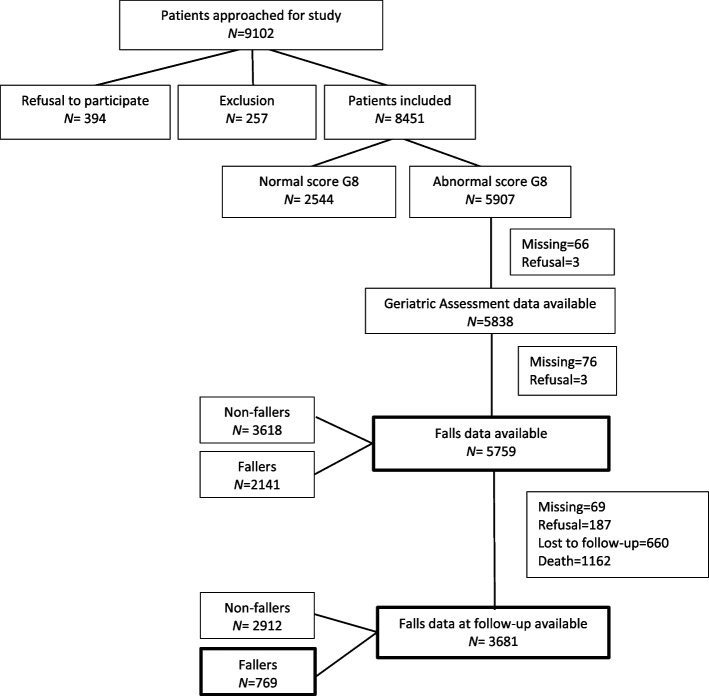
Flow-chart of patient selection

The patient flow-chart is presented in Fig. 1. We approached 9102 patients to participate in this study. Of these patients, 394 refused to participate and 257 did not meet the inclusion criteria resulting in 8451 included patients. We further selected patients based on an abnormal G8 score and the availability of GA data including fall-related data. This resulted in 5759 patients whose baseline falls history data were available. After approximately 3 months follow-up, fall-related data of 3681 patients were available.

Patient, socio-demographic and clinical characteristics are listed in Table 1. Of the included patients, 54.5% was female (*n* = 3133), and the median age was 80 years old (range 70–101). Most of the patients (77.9%) had a new diagnosis at inclusion, and 90.6% had a solid tumor. Comorbidity was present in 74.0%, and polypharmacy in 63.6% of the patients. Most patients lived at home, either alone (36.0%) or with a partner/family (55.2%) and 53.2% of the patients had professional home care.

**Table 1:** Baseline patient, socio-demographic and clinical characteristics and geriatric assessment data

		Patients with data on falls at baseline available (***n*** = 5759)		Patients with data on falls at follow-up available (***n*** = 3681)	
**Variable**	**Operationalization**	***N*** **(%)**	**95% CI**^**a**^	***N*** **(%)**	**95% CI**^**a**^
**Age (years)**	70–74	1212 (21.0)	19.99–22.10	815 (22.1)	20.80–23.48
	75–79	1556 (27.0)	25.87–28.17	1007 (27.4)	25.92–28.80
	80–84	1668 (29.0)	27.79–30.14	1038 (28.2)	26.74–29.65
	≥85	1323 (23.0)	21.87–24.04	821 (22.3)	20.96–23.65
	Median	80.0		80.0	
	Range	70–101		70–100	
**Sex**	Male	2626 (45.6)	44.31–46.88	1601 (43.5)	41.89–45.10
	Female	3133 (54.4)	53.12–55.69	2080 (56.5)	54.90–58.11
**Diagnosis general**	Solid tumor/Carcinoma	5218 (90.6)	89.85–91.36	3352 (91.1)	90.14–91.98
	Hematologic malignancy	541 (9.4)	8.64–10.15	329 (8.9)	8.02–9.86
**Diagnosis specific**	New diagnosis	4488 (77.9)	76.86–79.00	2907 (79.0)	77.66–80.29
	Relapse/ Progression	1271 (22.1)	21.00–23.14	774 (21.0)	19.71–22.34
**CCI**^a^	Score 0	1486 (26.0)	24.90–27.18	989 (27.0)	25.58–28.46
	Score ≥ 1	4220 (74.0)	72.82–75.10	2671 (73.0)	71.54–74.42
	Missing	53		21	
**Polypharmacy**^a^	Number 0–4	2052 (36.4)	35.12–37.63	1355 (37.3)	35.71–38.86
	Number ≥ 5	3589 (63.6)	62.37–64.88	2279 (62.7)	61.14–64.29
	Missing	118		47	
**ECOG-PS**	Score 0–1	2915 (50.6)	49.32–51.91	2098 (57.0)	55.40–58.60
	Score 2–4	2844 (49.4)	49.32–51.91	1583 (43.0)	41.40–44.60
**Living situation**^a^	Living alone	2070 (36.0)	34.70–37.18	1307 (35.5)	33.96–37.05
	Living with others	3179 (55.2)	53.92–56.49	2080 (56.5)	54.90–58.11
	Other	508 (8.8)	8.12–9.59	294 (8.0)	7.11–8.86
	Missing	2		0	
**Professional home care** ^a^	No	2694 (46.8)	45.55–48.13	1696 (46.1)	44.51–47.72
	Yes	3057 (53.2)	51.87–54.45	1982 (53.9)	52.28–55.50
	Missing	8		3	
**Geriatric domain**					
**FS: ADL (6–24)**	Independent: score 6	2329 (40.5)	39.1–41.7	1623 (44.1)	42.5–45.7
	Dependent: score ≥ 7	3430 (59.5)	58.0–61.0	2058 (55.9)	54.0–58.0
**FS: IADL** ^a^ **(0–5 (male)/8(female))**	Independent: score 8 (female) or 5 (male)	1817 (31.8)	30.3–32.7	1267 (34.4)	33.0–36.0
	Dependent: score < 8 or 5	3902 (68.2)	67.0–69.0	2401 (65.6)	64.0–67.0
	Missing	40		13	
**Pain (VAS) (0–10)** ^a^	No pain (score 0)	2725 (48.3)	47.0–49.6	1810 (50.1)	48.4–51.7
	Mild pain (score 0.5–3)	1086 (19.3)	18.2–20.3	717 (19.8)	18.5–21.1
	Severe (score 3.5–10)	1828 (32.4)	31.2–33.6	1087 (30.1)	28.6–31.6
	Missing	120		67	
**Fatigue (VAS) (0–10)** ^a^	No fatigue (score 0)	1261 (22.7)	21.6–23.8	937 (26.3)	24.8–27.7
	Presence of fatigue (score 0.5–10)	4299 (77.3)	76.0–78.0	2632 (73.5)	72.0–75.0
	Missing	199		112	
**Cognition (MMSE) (0–30)** ^a^	Score ≥ 24 = normal cognition	3942 (76.9)	75.7–78.1	2686 (80.1)	78.8–81.5
	Score 18–23 = mild cognitive impairment	831 (16.2)	15.2–17.2	495 (14.8)	13.6–16.0
	Score ≤ 17 = severe cognitive impairment	353 (6.9)	6.2–7.6	170 (5.1)	4.3–5.8
	Missing	633		330	
**Depression (GDS) (0–15)** ^a^	Score 0–4 = not at risk for depression	3301(62.8)	61.5–64.1	2297 (66.8)	65.3–68.4
	Score 5–15 = at risk for depression	1954 (37.2)	36.0–38.0	1140 (33.2)	32.0–35.0
	Missing	504		244	
**Nutrition (MNA-SF) (0–14)** ^a^	Normal nutritional status: score 12–14	1028 (17.9)	16.9–18.9	760 (20.7)	19.4–22.0
	Risk of malnutrition: score 8–11	2986 (52.0)	50.7–53.3	2022 (55.1)	53.4–56.6
	Malnourished: score 0–7	1728 (30.1)	28.9–31.3	891 (24.2)	22.9–25.6
	Missing	17		8	

^a^In case of missings, calculation of the percentages and 95% CI were made by subtracting the missings from the denominator

Abbreviations: *CCI* Charlson Comorbidity Index, *ECOG-PS* Eastern Cooperative Oncology Group – Performance Status, *FS* functional status, *ADL* Activities of Daily Living, *IADL* Instrumental Activities of Daily Living, *VAS* Visual Analogue Scale, *MMSE* Mini Mental State Examination, *GDS* Geriatric Depression Scale, *MNA-SF* Mini Nutritional Assessment – Short Form

### Geriatric assessment

Results of the GA are shown in Table 1. More than half of the patients showed a functional dependency on ADL (59.5%) and IADL (68.2%). The greatest clinical problems were fatigue (77.3%), and being at risk of malnutrition or malnourished (82%). A mild to severe pain was reported by 51.6% of the patients. Cognitive impairment was detected in 23.1% of the patients and 37.2% of the patients were at risk for depression.

### Incidence of falls, fall-related injuries and fear of falling at baseline and at follow-up

**Fig. 2:**
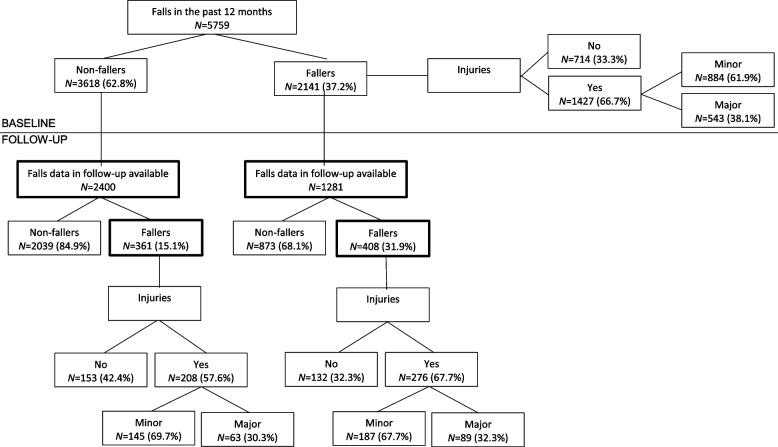
Overview of falls with fall-related injuries (minor + major). Minor injuries were scrapes and scratches, bruises, and superficial wounds that required no or minimal medical assistance. Major injuries were sprains, severe soft-tissue bruises, severe head injuries, distortion or dislocation of the joints, contusions, lacerations, loss of consciousness, and fractures

At baseline, 2141 (37.2%) patients reported at least one fall in the past 12 months before inclusion in the study, and 1427 patients (66.7%) sustained an injury (minor (61.9%); major (38.1%)) (see Table 2; Fig. 2).

**Table 2:** Results of fall-related data at baseline and follow-up

		Patients with data on falls history available (related to 1 year before inclusion) ***N*** = 5759^**a**^		Patients with data on falls in follow-up period available ***N*** = 3681^**a**^			
				Baseline data (related to 1 year before inclusion)		Follow-up data (related to follow-up period)	
		***N*** (%)	CI 95%	***N*** (%)	CI 95%	***N*** (%)	CI 95%
**Falls**	Non-fallers	3618 (62.8)	61.6–64.1	2400 (65.2)	63.7–66.7	2912 (79.1)	77.8–80.4
	Fallers	2141 (37.2)	35.9–38.4	1281 (34.8)	33.3–36.3	769 (20.9)	19.6–22.2
	• Single fallers	1117 (19.4)	18.4–20.4	686 (18.6)	17.4–19.9	465 (12.6)	11.6–13.7
	• Recurrent fallers	911 (15.8)	14.9–16.8	537 (14.6)	13.4–15.7	289 (7.9)	6.9–8.6
	• Unknown^b^	113 (2.0)	1.6–2.3	58 (1.6)	1.2–2.0	15 (0.4)	0.2–0.6
**Fall-related injuries**^**c**^	Non-fallers	3618 (62.8)	61.6–64.1	2400 (65.2)	63.7–66.7	2912 (79.1)	77.8–80.4
	Fallers without injuries	714 (12.4)	11.5–13.2	419 (11.4)	10.4–12.4	285 (7.7)	6.9–8.6
	Fallers with injuries	1427 (24.8)	23.7–25.9	862 (23.4)	22.0–24.8	484 (13.1)	12.1–14.2
	Fallers with minor injuries	884 (15.3)	14.4–16.3	536 (14.6)	13.4–15.7	332 (9.0)	8.1–9.9
	Fallers with major injuries	543 (9.4)	8.7–10.2	326 (8.9)	7.9–9.8	152 (4.1)	3.5–4.8
**Falls with fall-related injuries**	Non-fallers	3618 (62.8)	61.6–64.1	2400 (65.2)	63.7–66.7	2912 (79.1)	77.8–80.4
	Single fallers without injuries	395 (6.9)	6.2–7.5	241 (6.5)	5.7–7.3	179 (4.9)	4.2–5.6
	Single fallers with minor injuries	431 (7.5)	6.8–8.2	265 (7.2)	6.4–8.0	195 (5.3)	4.6–6.0
	Single fallers with major injuries	291 (5.1)	4.5–5.6	180 (4.9)	4.2–5.6	91 (2.5)	2.0–3.0
	Recurrent fallers without injuries	263 (4.6)	4.0–5.1	150 (4.1)	3.4–4.7	98 (2.7)	2.1–3.2
	Recurrent fallers with minor injuries	415 (7.2)	6.5–7.9	250 (6.8)	6.0–7.6	134 (3.6)	3.0–4.2
	Recurrent fallers with major injuries	233 (4.0)	3.5–4.6	137 (3.7)	3.1–4.3	57 (1.5)	1.1–1.9
	Unknown^b^	113 (2.0)	1.6–2.3	58 (1.6)	1.2–2.0	15 (0.4)	0.2–0.6
**Fear of falling**	Never	3015 (52.6)	51.3–53.9	2027 (55.3)	53.7–56.9	1631 (44.4)	42.8–46.0
	Sometimes	1256 (21.9)	20.8–23.0	773 (21.1)	19.8–22.4	1170 (31.9)	30.3–33.4
	Often	650 (11.3)	10.5–12.2	404 (11.0)	10.0–12.0	418 (11.4)	10.4–12.4
	Always	810 (14.1)	13.2–15.0	460 (12.6)	11.5–13.6	454 (12.4)	11.3–13.4
	Missing	28		17		8	

^a^In case of missings, calculation of the percentages and the 95% CI were made by subtracting the missings from the denominator

^b^Unknown: patients that experienced a fall but not known if it was a single fall or recurrent falls

^c^Minor injuries were scrapes and scratches, bruises, and superficial wounds that required no or minimal medical assistance. Major injuries were sprains, severe soft-tissue bruises, severe head injuries, distortion or dislocation of the joints, contusions, lacerations, loss of consciousness, and fractures

**Abbreviations**: *CI* confidence interval

The follow-up period of approximately 3 months had an average of 89 days with a standard deviation of 20 days.

During this follow-up period, 769 patients (20.9%) reported a fall of whom 289 (37.6%) fell more than once. The fall risk during follow-up was significantly higher in patients with falls history in the past 12 months compared to those without falls history (31.9% versus 15.1% respectively, *p* < 0.0001) (see Table 2; Fig. 2).

A fall-related injury was reported by 484 patients (62.9% of the fallers) of which 332 patients (68.6%) reported a minor injury and 152 patients (31.4%) reported a major injury (see Fig. 2).

Fear of falling was reported in 47.4% of the patients at baseline and in 55.6% of the patients at follow-up (see Table 2).

### Univariable and multivariable baseline predictors of falls at follow-up

Univariable predictive baseline factors for falls during the follow-up period were: age (*p*-value = 0.005), sex (*p*-value = 0.036), CCI (*p*-value = 0.028), polypharmacy (*p*-value = <.0001), ECOG-PS (*p*-value = <.0001), professional home care (*p*-value = 0.001), functional status (FS) measured by ADL (*p*-value = <.0001) and IADL (*p*-value = <.0001), falls history in the past 12 months (*p*-value = <.0001), fear of falling (*p*-value = <.0001), MMSE (*p*-value = <.0001), GDS (*p*-value = 0.001) and MNA-SF (*p*-value = 0.023) (see Table 3).

**Table 3:** Univariable baseline predictors of falls at follow-up

					***P***-value
**Covariate**		**Patients with data on falls in follow-up period available (*n*** **= 3681)**	**Non-fallers (*n*** **= 2912)**	**Fallers (*n*** **= 769)**	**Non- fallers vs. fallers**
**Age**	70–74	815 (22.1)	676 (23.2)	139 (18.1)	**0.005**
	75–79	1007 (27.4)	784 (26.9)	223 (29.0)	
	80–84	1038 (28.2)	827 (28.4)	211 (24.4)	
	≥85	821 (22.3)	625 (21.5)	196 (25.5)	
**Sex**	Female	2080 (56.5)	1672 (57,4)	408 (53.1)	**0.036**
	Male	1601 (43.5)	1240 (42, 5)	361 (46.9)	
**Diagnosis General**	Solid tumor	3352 (91.1)	2665 (91.5)	687 (89.3)	0.065
	Hematologic malignancy	329 (8.9)	247 (8.5)	82 (10.7)	
**Diagnosis Specific**	New diagnosis	2907 (79.0)	2303 (79.1)	604 (78.5)	0.743
	Relapse/Progression	774 (21.0)	609 (20.9)	165 (21.5)	
**CCI**^**a**^	No comorbidities (0)	989 (27.0)	806 (27.8)	183 (23.9)	**0.028**
	Comorbidities (≥1)	2671 (73.0)	2089 (72.2)	582 (76.1)	
**Polypharmacy**^**a**^	No polypharmacy (0–4)	1355 (37.3)	1124 (39.1)	231 (30.3)	**< 0.0001**
	Polypharmacy (≥5)	2279 (62.7)	1748 (60.9)	531 (69.7)	
**ECOG-PS**	Score 0–1	2098 (57.0)	1732 (59.5)	366 (47.6)	**< 0.0001**
	Score 2–4	1583 (43.0)	1180 (40.5)	403 (52.4)	
**Living situation**	Not living alone	2374 (64.5)	1889 (64.9)	485 (63.1)	0.354
	Living alone	1307 (35.5)	1023 (35.1)	284 (36.9)	
**Professional home care**^**a**^	No	1696 (46.1)	1382 (47.5)	314 (40.9)	**0.001**
	Yes	1982 (53.9)	1528 (52.5)	454 (59.1)	
**FS: ADL**	Independent (6)	1623 (44.1)	1368 (47.0)	255 (33.2)	**< 0.0001**
	Dependent (> 6)	2058 (55.9)	1544 (53.0)	514 (66.8)	
**FS: IADL**^**a**^	Independent (5(male)/8(female))	1267 (34.5)	1072 (36.9)	195 (25.5)	**< 0.0001**
	Dependent (< 5(male)/8(female)	2401 (65.5)	1831 (63.1)	570 (74.5)	
**Falls history in the past 12 months**	No falls	2400 (65.2)	2039 (70.0)	361 (46.9)	**< 0.0001**
	Falls	1281 (34.8)	873 (30.0)	408 (53.1)	
**Fear of falling**^**a**^	Never	2027 (55.3)	1664 (57.4)	363 (47.3)	**< 0.0001**
	Sometimes / often / always	1637 (44.7)	1233 (42.6)	404 (52.7)	
**VAS for fatigue**^**a**^	No fatigue (0)	937 (26.3)	756 (26.7)	181 (24.4)	0.193
	Presence of fatigue (≥1)	2632 (73.7)	2071 (73.3)	561 (75.6)	
**VAS for pain**^**a**^	No pain (0)	1810 (50.1)	1453 (50.8)	357 (47.4)	0.099
	Presence of pain (≥1)	1804 (49.9)	1408 (49.2)	396 (52.6)	
**MMSE**^**a**^	Normal cognition (≥24)	2686 (80.2)	2184 (82.1)	502 (72.5)	**< 0.0001**
	Cognitive impairment (< 24)	665 (19.8)	475 (17.9)	190 (27.5)	
**GDS**^**a**^	Not at risk for depression (< 5)	2297 (66.8)	1859 (68.2)	438 (61.4)	**0.001**
	At risk for depression (≥5)	1140 (33.2)	865 (31.8)	275 (38.6)	
**MNA-SF**^**a**^	Normal nutritional status (≥12)	760 (20.7)	624 (21.5)	136 (17.8)	**0.023**
	Risk of malnutrition/ malnourished (< 12)	2913 (79.3)	2284 (78.5)	629 (82.2)	

^a^In case of missings, calculation of the percentages was made by subtracting the missings from the denominator

Abbreviations: *FS* functional status, *ADL* Activities of Daily Living, *IADL* Instrumental Activities of Daily Living, *VAS* Visual Analogue Scale, *MMSE* Mini Mental State Examination, *GDS* Geriatric Depression Scale, *MNA-SF* Mini Nutritional Assessment – Short Form, *CCI* Charlson Comorbidity Index, *ECOG-PS* Eastern Cooperative Oncology Group – Performance Status

**Fig. 3:**
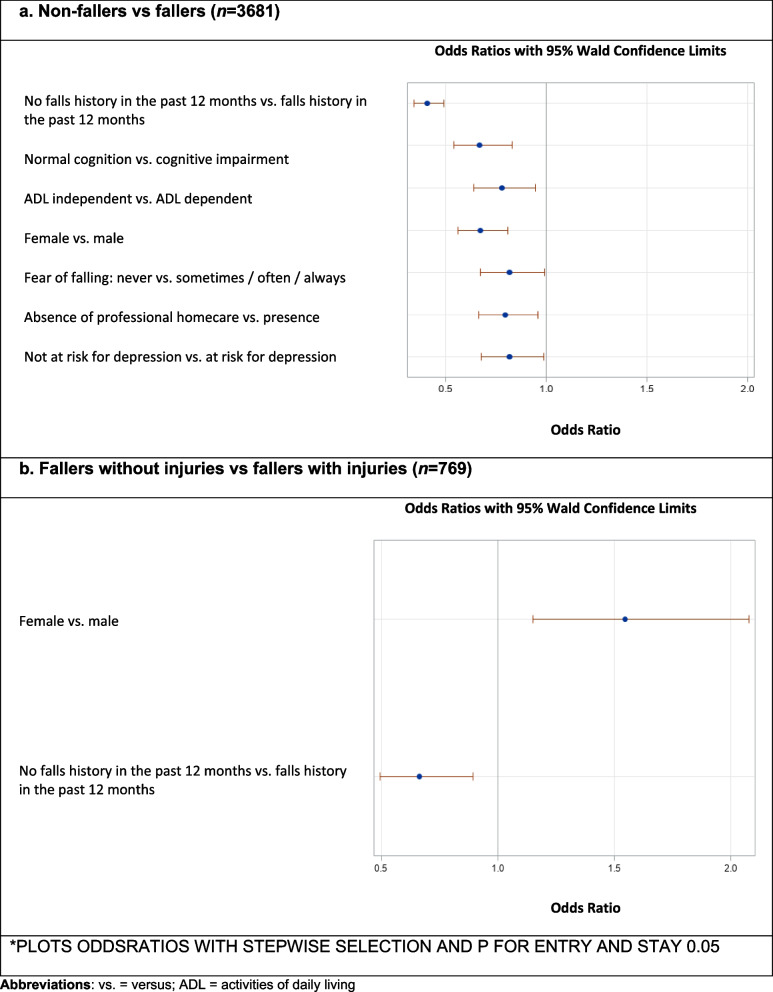
Multivariable baseline predictors for falls (≥1 fall) and fall-related injuries at follow-up

In multivariable regression analysis, falls during the follow-up period can be predicted significantly by presence of falls history in the past 12 months (OR: 0.41; 95%CI: 0.340–0.490), cognitive impairment measured by MMSE (OR: 0.70; 95%CI: 0.540–.0830), functional dependency measured by ADL (OR: 0.78; 95%CI: 0.639–.0947), male sex (OR: 0.67; 95%CI: 0.558–0.808), fear of falling (OR: 0.82; 95%CI: 0.673–0.989), use of professional homecare (OR: 0.80; 95%CI: 0.662–0.957), and a risk for depression measured by GDS-15 (OR: 0.82; 95%CI: 0.676–0.988) (see Fig. 3a).

There was no multicollinearity between the independent variables.

### Univariable and multivariable baseline predictors of fall-related injuries at follow-up

Both univariable and multivariable predictive factors for fall-related injuries at follow-up were female sex (*p*-value = 0.003; OR: 1.52; 95% CI: 1.095–2.097) and presence of a falls history in the past 12 months (*p*-value = 0.004; OR: 0.66; 95% CI: 0.474–0.908) (see Table 4; Fig. 3b).

**Table 4:** Univariable baseline predictors of fall-related injuries at follow-up

Covariate		Fallers (***n*** = 769) ***n*** (%)	Fallers without injury (ies) (***n*** = 285) ***n*** (%)	Fallers with injury (ies) (***n*** = 484) ***n*** (%)	Fallers without injuries vs fallers with injury (ies) ***p***-value
Age	70–74	139 (18.1)	57 (20.0)	82 (16.9)	0.612
	75–79	223 (29.0)	81 (28.4)	142 (29.3)	
	80–84	211 (24.4)	80 (28.1)	131 (27.1)	
	≥85	196 (25.5)	67 (23.5)	129 (26.7)	
Sex	Female	408 (53.1)	131 (46.0)	277 (57.2)	**0.003**
	Male	361 (46.9)	154 (54.0)	207 (42.8)	
Diagnosis General	Solid tumor	687 (89.3)	256 (89.8)	431 (89.0)	0.762
	Hematologic malignancy	82 (10.7)	29 (10.2)	53 (11.0)	
Diagnosis Specific	New diagnosis	604 (78.5)	226 (79.3)	378 (78.1)	0.772
	Progression / Relapse	165 (21.5)	59 (20.7)	106 (21.9)	
CCI^a^	No comorbidities (0)	183 (23.9)	68 (24.2)	115 (23.8)	0.950
	Comorbidities (≥1)	582 (76.1)	213 (75.8)	369 (76.2)	
Polypharmacy^a^	No polypharmacy (0–4)	231 (30.3)	88 (31.0)	143 (29.9)	0.715
	Polypharmacy (≥5)	531 (69.7)	196 (69.0)	336 (70.1)	
ECOG-PS	Score 0–1	366 (47.6)	137 (48.1)	229 (47.3)	0.709
	Score 2–4	403 (52.4)	148 (51.9)	255 (52.7)	
Living situation	Not living alone	485 (63.1)	187 (65.6)	298 (61.6)	0.248
	Living alone	284 (36.9)	98 (34.4)	186 (38.4)	
Professional home care^a^	No	314 (40.9)	124 (43.5)	190 (39.3)	0.239
	Yes	454 (59.1)	161 (56.5)	293 (60.7)	
FS: ADL	Independent (6)	255 (33.2)	101 (35.4)	154 (31.8)	0.241
	Dependent (> 6)	514 (66.8)	184 (64.6)	330 (68.2)	
FS: IADL^a^	Independent (5(male)/8(female))	195 (25.5)	73 (25.7)	122 (25.4)	0.801
	Dependent (< 5(male)/8(female)	570 (74.5)	211 (74.3)	359 (74.6)	
Falls history in the past 12 months	No falls	361 (46.9)	153 (53.7)	208 (43.0)	**0.004**
	Falls	408 (53.1)	132 (46.3)	276 (57.0)	
Fear of falling^a^	Never	363 (47.3)	132 (46.6)	231 (47.7)	0.772
	Sometimes / often / always	404 (52.7)	151 (53.4)	253 (52.3)	
VAS for fatigue^a^	No fatigue (0)	181 (24.4)	69 (25.1)	112 (24.0)	0.742
	Presence of fatigue (≥1)	561 (75.6)	206 (74.9)	355 (76.0)	
VAS for pain^a^	No pain (0)	357 (47.4)	131 (46.6)	226 (47.9)	0.724
	Pain (≥1)	396 (52.6)	150 (53.4)	246 (52.1)	
MMSE^a^	Normal cognition (≥24)	502 (72.5)	183 (72.0)	319 (72.8)	0.997
	Cognitive impairment (< 24)	190 (27.5)	71 (28.0)	119 (27.2)	
GDS^a^	Not at risk for depression (< 5)	438 (61.4)	167 (63.3)	271 (60.4)	0.433
	At risk for depression (≥5)	275 (38.6)	97 (36.7)	178 (39.6)	
MNA-SF^a^	Normal nutritional status (≥12)	136 (17.8)	46 (16.3)	90 (18.6)	0.435
	Risk of malnutrition/ malnourished (< 12)	629 (82.2)	236 (83.7)	393 (81.4)	

^a^In case of missings, calculation of the percentages was made by subtracting the missings from the denominator

Abbreviations: *ADL* Activities of Daily Living, *IADL* Instrumental Activities of Daily Living, *CCI* Charlson Comorbidity Index, *VAS* Visual Analogue Scale, *MMSE* Mini Mental State Examination, *GDS* Geriatric Depression Scale, *MNA-SF* Mini Nutritional Assessment-Short Form, *ECOG-PS* Eastern Cooperative Oncology Group Performance

## Discussion

This study focused on the incidence and predictive factors for falls and fall-related injuries in older patients with cancer with a frailty profile, based on abnormal score on the G8 screening tool. Almost 4 out of 10 in the past 12 months and 1 out of 5 at approximately 3 months of follow-up had at least one fall, respectively. Almost 7 out of 10 fallers experienced fall-related injuries and more than half suffered from fear of falling at follow-up. The following predictive factors seem to play an important role for both falls and fall-related injuries in this population: sex and falls history in the past 12 months. Male patients had a higher risk for falls during follow-up and in contrary the risk for fall-related injuries was higher in female patients. Several other components were also predictive factors for falls: risk for depression, cognitive impairment, dependency in ADL, fear of falling and use of professional home care.

The amount of frail older patients with cancer experiencing a fall history seems to be congruent with figures found by Zhang et al. showing a fall rate of 38.5% in the past 6 months before inclusion in their study. In this study 53% of the included patients had a frailty profile, based on Fried’s criteria [11].

The fall incidence of 20.9% at follow-up in this study is somewhat higher compared with other studies in older patients with cancer. Vande Walle and Puts conclude a fall rate of 17.6% in a follow-up period of two to 3 months [9], and 18.7% in a follow up period of 6 months [40]; respectively. Both studies did not exclusively focus on patients with a frailty profile. The study of Vande Walle et al. described the presence of a frailty profile based on an abnormal result on the G8 screening as in our study, present in 74.4% of the patients, whereas the study of Puts et al. didn’t define if the patient had a frailty profile or not. Frailty indeed influences these figures; i.e. in an unplanned post hoc analysis of the current study we found a much lower incidence of 8.8% for falls at follow-up for non-frail patients (G8 score > 14; patients not included in this study because no baseline geriatric assessment data available). Furthermore, a study of Stone et al. concluded a much higher fall rate of 50.3% during a follow-up period of 6 months [41]. This might be due to the inclusion of people with metastatic or locoregionally advanced cancer and as a result negatively influencing the fall incidence.

Literature shows that the population of older persons with cancer has a higher risk of injuries which can be due to the characteristics that come with cancer, such as cancer treatment (e.g. chemotherapy), cancer stage, comorbidity, and osteoporosis [20, 42]. The number of injuries found in our study are somewhat comparable to those reported in the study of Vande Walle et al. (i.e. 62.0% experiencing fall-related injuries at two to 3 months follow-up) [9]. Two other studies in a population of older persons with cancer concluded an injury rate of 42 to 45%, but both studies didn’t report data related on a frailty profile [41, 43]. Thus, it seems that older persons with cancer and frailty profile have a higher risk of sustaining an injury after a fall than older persons of the general population with cancer.

Fear of falling is very common in older patients with cancer, as shown by the current study. Data on fear of falling in the older population with cancer is scarce and only a few studies reported some information on this topic [44]. One study of Sattar et al. (2019) reported a prevalence rate of fear of falling of 55% which is comparable with our results, but again data on a frailty profile are not available [38]. Prevalence rates in the general older population are varying a lot (e.g. between 3 and 85%) and this might be due to different methods and tools of measurements [45].

Falls history is described in the literature as the main predictive factor for falls and fall-related injuries, both in the general older population and the population of older patients with cancer [9, 11, 41]. This was confirmed in the current study. Overall, a higher risk for falls in females is reported more often in the general older population [46, 47]. However, our study shows that both male and female sex play an important role as predictive factor; e.g. males having a higher risk for falls than females during follow-up but females having a higher risk for fall-related injuries. Although more research is needed to explain this finding, behavioral (e.g. males taking more risk behavior and overestimating their true ability compared to females) and biological differences (e.g. females being more at risk for osteoporosis) might play a role in this [48]. Being at risk for depression and the presence of cognitive impairment are also predictive factors for falls in this study. Regarding the risk for depression, other research shows that depression increases fear of falling (high incidence in our study), which in turn increases the risk of a fall [11]. In addition our study shows that 23.1% of the older patients experience cognitive impairment. This is a high number compared to a percentage of 10.6%, found in another study [49], and may be due to the fact that this study includes patients with a frailty profile. Therefore, this high percentage of cognitive impairment could explain the high number of falls and fall-related injuries during follow-up. Indeed, cognitive impairment is a well-known fall risk factor [50]. ADL dependence is another predictor for falls during follow-up. A systematic review by Wildes et al. reported that an association exists between functional dependence measured by ADL and risk for falls in the community-dwelling population, and that this association remains present in older persons with cancer [14]. Another predictive factor for falls during follow-up in our study is fear of falling. Based on the literature in the general older population we know that previous falls can induce or increase fear of falling leading to reduced activities in daily living [51]. Decreased functionality can lead to an increased risk for falls, which was one of the main predictive factors for falls during follow-up in this study. Finally, the last predictive factor for falls during follow-up in this study was the presence of professional home care. This can possibly be explained by the fact that this study focused on older patients with a frailty profile who are possible more in need of professional homecare and therefore have a higher risk for falls.

Age, functional status measured by IADL, comorbidities, polypharmacy, ECOG-PS and nutritional status measured by MNA-SF were significant predictors in univariate analysis, but not significant in the multivariable analysis in this study. It is clear that several variables are in some way interconnected, and some studies withheld other significant variables after multivariable analysis [10, 11, 14, 20, 40]. Therefore, it is important for healthcare providers to be attentive to these problems also, as they play an important role in the overall health status of older persons with cancer.

A strength of this study is that the data set was a very large one, that included a representative picture on nearly all solid tumors and hematological malignancies seen in daily oncology practice. Another strength of this study is that it provides new information about the incidence of falls and fall-related injuries and associated predictive factors for falls and fall-related injuries in older persons with cancer and a frailty profile. The G8 tool was used to determine frailty in our study. This is a highly sensitive tool for the population of older persons with cancer [23]. The G8 tool provides a fast and reliable method to detect the persons who would benefit from more extensive GA.

### Limitations

The period of follow-up is approximately 3 months. This is a short period and more research with a longer follow-up period is needed. The patients in this study were asked about falls and fall-related injuries in the past 12 months and during the follow-up period. Using self-report for collecting these data without verification from electronic patient charts incorporates always a chance on recall bias. Especially since the percentage of patients with cognitive impairment is high, although the majority of these patients had a mild cognitive impairment. Integrating the caregiver of the patient can be helpful to address this concern in future research.

Secondly, although fear of falling was not a primary outcome parameter within this study, the integration of a validated instrument like the Falls Efficacy Scale – International (FES-I) is recommended to evaluate fear of falling in future research [39, 52].

Finally, this was a preplanned secondary data analysis, and variables shown to be significant in previous studies, such as type of medication (e.g. benzodiazepines, antidepressants, and anti-psychotics) [53, 54] were not included in the data base of this study. Other variables that were not included were the circumstances of a fall, time point of a fall, impact of the incidence of falls and fall-related injuries on subsequent cancer treatment and efficacious interventions for fall prevention [21]. Further research on these aspects is therefore highly recommended.

## Conclusion

This study shows that falls and fall-related injuries occur frequently in older persons with cancer and a frailty profile and that fear of falling is common in this patient population. Systematic fall screening when taking care for frail older patients with cancer in daily oncology practice is needed and needs to be integrated within the GA. Sex and falls history are common predictors for both falls and fall-related injuries. Other predictive factors for falls were risk for depression, cognitive impairment, dependency in ADL, fear of falling and use of professional home care. Designing and integrating GA-tailored falls prevention interventions in daily care of frail older patients with cancer are highly recommended.

## Data Availability

The datasets used and/or analyzed during the current study are available from the corresponding author on reasonable request.

## Abbreviations

ADL: Activities of Daily Living
CCI: Charlson Comorbidity index
ECOG-PS: Eastern Cooperative Oncology Group - Performance Status
FS: Functional status
GA: Geriatric assessment
GDS: Geriatric depression scale
IADL: Instrumental Activities of Daily Living
MMSE: Mini mental state examination
MNA-SF: Mini nutritional assessment – short form
ProFouND: The Prevention of Falls Network for Dissemination
USA: United States of America
VAS: Visual analogue scale
VIF: Variance inflation factors
WHO: World health organization

## References

[1] Lamb SE, Jørstad-Stein EC, Hauer K, Becker C (2005). Development of a common outcome data set for fall injury prevention trials: the prevention of falls network Europe consensus. J Am Geriatr Soc.

[2] Hauer K, Lamb SE, Jorstad EC, Todd C, Becker C (2006). Systematic review of definitions and methods of measuring falls in randomised controlled fall prevention trials. Age Ageing.

[3] WHO Global Report on Falls Prevention in Older Age. https://www.who.int/ageing/publications/Falls_prevention7March.pdf?ua=1. Accessed 23 Apr 2021.

[4] Tinetti ME, Speechley M, Ginter SF (1988). Risk factors for falls among elderly persons living in the community. N Engl J Med.

[5] Rubenstein LZ (2006). Falls in older people: epidemiology, risk factors and strategies for prevention. Age Ageing.

[6] Florence CS, Bergen G, Atherly A, Burns E, Stevens J, Drake C (2018). Medical costs of fatal and nonfatal falls in older adults. J Am Geriatr Soc.

[7] Smith BD, Smith GL, Hurria A, Hortobagyi GN, Buchholz TA (2009). Future of cancer incidence in the United States: burdens upon an aging, changing nation. J Clin Oncol.

[8] World Cancer Report: Cancer Research for Cancer Prevention. https://publications.iarc.fr/Non-Series-Publications/World-Cancer-Reports/World-Cancer-Report-Cancer-Research-For-Cancer-Prevention-2020. Accessed 23 Apr 2021.

[9] Vande Walle N, Kenis C, Heeren P (2014). Fall predictors in older cancer patients: a multicenter prospective study. BMC Geriatr.

[10] Wildes TM, Maggiore RJ, Tew WP (2018). Factors associated with falls in older adults with cancer: a validated model from the Cancer and aging research group. Support Care Cancer.

[11] Zhang X, Sun M, Liu S (2018). Risk factors for falls in older patients with cancer. BMJ Support Palliat Care.

[12] Mohile SG, Fan L, Reeve E (2011). Association of cancer with geriatric syndromes in older Medicare beneficiaries. J Clin Oncol.

[13] Spoelstra SL, Given BA, Schutte DL, Sikorskii A, You M, Given CW (2013). Do older adults with cancer fall more often? A comparative analysis of falls in those with and without cancer. Oncol Nurs Forum.

[14] Wildes TM, Dua P, Fowler SA (2015). Systematic review of falls in older adults with cancer. J Geriatr Oncol.

[15] Clegg A, Young J, Iliffe S, Rikkert MO, Rockwood K (2013). Frailty in elderly people. Lancet.

[16] Ferrucci L, Guralnik JM, Studenski S, Fried LP, Cutler GB, Walston JD (2004). Designing randomized, controlled trials aimed at preventing or delaying functional decline and disability in frail, older persons: a consensus report. J Am Geriatr Soc.

[17] Mohile SG, Xian Y, Dale W (2009). Association of a cancer diagnosis with vulnerability and frailty in older Medicare beneficiaries. J Natl Cancer Inst.

[18] Lang PO, Michel JP, Zekry D (2009). Frailty syndrome: a transitional state in a dynamic process. Gerontology.

[19] Reiser E, Pötsch N, Seebacher V (2021). Impact of frailty on the management of patients with gynecological cancer aged 80 years and older. Arch Gynecol Obstet.

[20] Sattar S, Alibhai SM, Spoelstra SL, Fazelzad R, Puts MT (2016). Falls in older adults with cancer: a systematic review of prevalence, injurious falls, and impact on cancer treatment. Support Care Cancer.

[21] Sattar S, Haase K, Kuster S (2021). Falls in older adults with cancer: an updated systematic review of prevalence, injurious falls, and impact on cancer treatment. Support Care Cancer.

[22] Kenis C, Decoster L, Flamaing J (2018). Adherence to geriatric assessment-based recommendations in older patients with cancer: a multicenter prospective cohort study in Belgium. Ann Oncol.

[23] Liuu E, Canouï-Poitrine F, Tournigand C (2014). Accuracy of the G-8 geriatric-oncology screening tool for identifying vulnerable elderly patients with cancer according to tumour site: the ELCAPA-02 study. J Geriatr Oncol.

[24] Charlson ME, Pompei P, Ales KL, MacKenzie CR (1987). A new method of classifying prognostic comorbidity in longitudinal studies: development and validation. J Chronic Dis.

[25] Sharma M, Loh KP, Nightingale G, Mohile SG, Holmes HM (2016). Polypharmacy and potentially inappropriate medication use in geriatric oncology. J Geriatr Oncol.

[26] Oken MM, Creech RH, Tormey DC (1982). Toxicity and response criteria of the eastern cooperative oncology group. Am J Clin Oncol.

[27] Katz S, Ford AB, Moskowitz RW, Jackson BA, Jaffe MW (1963). Studies of illness in the aged. The index of ADL: a standardized measure of biological and psychosocial function. JAMA.

[28] Lawton MP, Brody EM (1969). Assessment of older people: self-maintaining and instrumental activities of daily living. Gerontologist.

[29] Collins SL, Moore RA, McQuay HJ (1997). The visual analogue pain intensity scale: what is moderate pain in millimetres?. Pain.

[30] Lee KA, Hicks G, Nino-Murcia G (1991). Validity and reliability of a scale to assess fatigue. Psychiatry Res.

[31] Folstein MF, Folstein SE, McHugh PR (1975). "Mini-mental state". A practical method for grading the cognitive state of patients for the clinician. J Psychiatr Res.

[32] Yesavage JA, Brink TL, Rose TL (1982). Development and validation of a geriatric depression screening scale: a preliminary report. J Psychiatr Res.

[33] Guigoz Y, Vellas B (1999). The Mini nutritional assessment (MNA) for grading the nutritional state of elderly patients: presentation of the MNA, history and validation. Nestle Nutr Workshop Ser Clin Perform Programme.

[34] Rubenstein LZ, Harker JO, Salvà A, Guigoz Y, Vellas B (2001). Screening for undernutrition in geriatric practice: developing the short-form mini-nutritional assessment (MNA-SF). J Gerontol A Biol Sci Med Sci.

[35] O'Halloran AM, Pénard N, Galli A, Fan CW, Robertson IH, Kenny RA (2011). Falls and falls efficacy: the role of sustained attention in older adults. BMC Geriatr.

[36] Milisen K, Coussement J, Flamaing J (2012). Fall prediction according to nurses' clinical judgment: differences between medical, surgical, and geriatric wards. J Am Geriatr Soc.

[37] Milisen K, Staelens N, Schwendimann R (2007). Fall prediction in inpatients by bedside nurses using the St. Thomas's risk assessment tool in falling elderly inpatients (STRATIFY) instrument: a multicenter study. J Am Geriatr Soc.

[38] Sattar S, Spoelstra SL, Alibhai SMH, Puts MTE (2019). Circumstances of falls and fear of falling in community-dwelling older adults with cancer: results from a mixed-methods study. J Geriatr Oncol.

[39] Sammels M, Vandesande J, Vlaeyen E, Peerlinck K, Milisen K (2014). Falling and fall risk factors in adults with haemophilia: an exploratory study. Haemophilia.

[40] Puts MT, Monette J, Girre V (2013). The fall rate of older community-dwelling cancer patients. Support Care Cancer.

[41] Stone CA, Lawlor PG, Savva GM, Bennett K, Kenny RA (2012). Prospective study of falls and risk factors for falls in adults with advanced cancer. J Clin Oncol.

[42] Edwards BJ, Sun M, Zhang X (2018). Fractures frequently occur in older cancer patients: the MD Anderson Cancer Center experience. Support Care Cancer.

[43] Sattar S, Alibhai SMH, Spoelstra SL, Puts MTE (2019). The assessment, management, and reporting of falls, and the impact of falls on cancer treatment in community-dwelling older patients receiving cancer treatment: results from a mixed-methods study. J Geriatr Oncol.

[44] Aburub AS, Phillips SP, Curcio CL, Guerra RO, Auais M (2020). Fear of falling in community-dwelling older adults diagnosed with cancer: a report from the international mobility in aging study (IMIAS). J Geriatr Oncol.

[45] Scheffer AC, Schuurmans MJ, van Dijk N, van der Hooft T, de Rooij SE (2008). Fear of falling: measurement strategy, prevalence, risk factors and consequences among older persons. Age Ageing.

[46] Campbell AJ, Spears GF, Borrie MJ (1990). Examination by logistic regression modelling of the variables which increase the relative risk of elderly women falling compared to elderly men. J Clin Epidemiol.

[47] Ward PR, Wong MD, Moore R, Naeim A (2014). Fall-related injuries in elderly cancer patients treated with neurotoxic chemotherapy: a retrospective cohort study. J Geriatr Oncol.

[48] Jehu DA, Davis JC, Barha CK (2022). Sex differences in subsequent falls and falls risk: a prospective cohort study in older adults. Gerontology.

[49] Kenis C, Decoster L, Van Puyvelde K (2014). Performance of two geriatric screening tools in older patients with cancer. J Clin Oncol.

[50] Muir SW, Gopaul K, Montero Odasso MM (2012). The role of cognitive impairment in fall risk among older adults: a systematic review and meta-analysis. Age Ageing.

[51] Murphy SL, Dubin JA, Gill TM (2003). The development of fear of falling among community-living older women: predisposing factors and subsequent fall events. J Gerontol A Biol Sci Med Sci.

[52] Yardley L, Beyer N, Hauer K, Kempen G, Piot-Ziegler C, Todd C (2005). Development and initial validation of the falls efficacy scale-international (FES-I). Age Ageing.

[53] Turner JP, Shakib S, Singhal N (2014). Prevalence and factors associated with polypharmacy in older people with cancer. Support Care Cancer.

[54] de Jong MR, Van der Elst M, Hartholt KA (2013). Drug-related falls in older patients: implicated drugs, consequences, and possible prevention strategies. Ther Adv Drug Saf.

